# P-1326. Trends in Transgender Sexual Health: New Findings from Syphilis Cases

**DOI:** 10.1093/ofid/ofae631.1504

**Published:** 2025-01-29

**Authors:** Sage W R Geher, Adam Spivak, Erika A Sullivan, Tiffany F Ho

**Affiliations:** University of Utah, School of Medicine, Salt Lake City, Utah; University of Utah School of Medicine, Salt Lake City, UT; University of Utah, Salt Lake City, Utah; University of Utah, Salt Lake City, Utah

## Abstract

**Background:**

Syphilis has been on an alarming rise with worldwide cases in excess of 7 million in 2020 and US case rates tripling over the last 20 years. Sexually transmitted infections disproportionally impact sexually minoritized groups, including transgender and gender diverse (TGD) adults. Yet, syphilis rates among TGD people are relatively understudied. We sought to investigate syphilis trends specifically in the TGD community.

**Methods:**

We performed a retrospective chart review and identified 33 TGD adults diagnosed with syphilis, from a total cohort population of 1,072 transgender persons tested for syphilis from 2010-2023. Persons diagnosed with syphilis were identified by one of the following criteria: International Classification of Diseases-10 (ICD) code, positive treponemal test (Tp), or positive non-treponemal test (NT). Diagnoses were further delineated into individual cases of syphilis to account for re-infections. True cases were defined as having positive Tp and NT, presumed positive as positive Tp or NT, and false positives as having a negative Tp or NT. Variables such as syphilis incidence and prevalence were calculated. Various qualitative aspects of disease progression and case presentation were analyzed via manual chart review.

**Results:**

We identified 22 true cases, 8 presumed positives, and 12 false positives. Syphilis incidence was 2.79% and lifetime prevalence of 1.95%. While the number of syphilis tests increased per year, test positivity rates remained fairly constant at 3-4%. The vast majority of cases presented as primary syphilis (72%), and were most often completely asymptomatic (59%), with chancre (24%) and unexplained headache (14%) being the most common symptoms across all stages. There was only one case (3%) of confirmed neurosyphilis, which is higher than the national incidence (0.8-1.7%). Titers at time of diagnosis spanned a large range of values and was not predictive of stage of presentation, nor was there a clear correlation in titer kinetics and response to treatment.

**Conclusion:**

With a relative paucity of investigations looking at syphilis rates in the TGD community, especially in the United States, understanding the epidemiology of this population could prove critical in prevention efforts.

**Disclosures:**

**All Authors**: No reported disclosures

